# Critical insights to COVID‐19 disease and potential treatments using a systems analysis approach that integrates physiology, pharmacology, and clinical pharmacology

**DOI:** 10.1002/prp2.918

**Published:** 2022-02-01

**Authors:** Jennifer H. Martin, Richard J. Head

**Affiliations:** ^1^ The University of Newcastle Hunter Medical Research Institute New Lambton New South Wales Australia; ^2^ Clinical and Health Sciences University of South Australia Adelaide South Australia Australia

The power of pharmacology and its application by way of clinical pharmacology to provide invaluable insights into COVID‐19 disease is brought home vividly when exploring the infectivity and pathophysiology of SARS‐COV‐2 in this pandemic. At its core, pharmacology utilizes and displays systems analysis to identify and correct aberrant physiology in disease or to provide much needed bridging intervention in a crisis. Pharmacology describes the impact of exogenous molecules upon living systems and to achieve this the discipline has over decades developed sophisticated measures to monitor absorption, distribution, excretion, and kinetics of interaction with the principal influenced tissues and receptors.

SARS‐COV‐2 is also an exogenous entity that displays some similar features as drugs of nasal or oral absorption, and which causes pathophysiologic responses in the human. Fortuitously, the application of spectacular advances in cell biology, structural biology, virology, and immunology to COVID‐19 disease has provided an invaluable background for this detailing of the pathogenesis of COVID‐19 from a pharmacological perspective. Employing well‐established fundamental principles of pharmacology in COVID‐19 disease adds significantly to our knowledge base of COVID‐19 disease. Additionally, as highlighted earlier^1^ it is imperative to understand the pathogenic response in COVID‐19 disease when evaluating potential therapeutic interventions. It has also been argued in this Journal that globally we have missed significant opportunities to develop pharmacologically sound, physiologically appropriate, affordable, and effective therapeutic options with which to treat people who are unable to access effective vaccines or in whom despite vaccination are vulnerable to even a mild immune response to COVID‐19.^2^, ^3^

The following thematic reviews specifically adopt a systems analysis approach using the pharmacological principles outlined above to further our insights into the role of SARS‐COV‐2 in this pandemic. The sequence of these thematic reviews deserves comment. They have been deliberately arranged in an order that tracks the time‐based development of the infection by SARS‐COV‐2 following its species jump to the human, the tropism associated with nasopharyngeal infection and in individuals with serious disease to the progression to a dysregulated inflammatory state and in selected individuals the progression to ‘Long COVID’ with its differential host response.

The Review by Head et al.^4^ focusses on the clash of two distinct evolutionary paths: that of the vulnerable complexity of the human with the lethal simplicity of SARS‐CoV‐2. In doing so it highlights the fundamental role played by thermodynamics in the spread of SARS‐COV‐2 from the infection of a single cell through to the global population. In essence the SARS‐CoV‐2 host‐disease interface is a complex system that can be viewed from a pharmacological perspective. COVID‐19 can be seen as a unique synchrony of viral surface change with Gibbs free energy liberation upon target binding that drives thermodynamic spontaneity. In this way the authors have provided a rational basis for the time course of the pathophysiology, for it is this binding that permits viral tropism and distribution across populations. It is necessary that this thermodynamic spontaneity is repeated endlessly and an initial single host cell infection with subsequent spreads to bystander cells and to tissues.

Head et al.^4^ also focused on the pivotal role of the mucosal layers in establishing a gradient with SARS‐CoV‐2 from the airway interface to the epithelial cell surface. It is an area that would appear important for designing any interventions to sequester this virus. Of major significance is the proposal that upper respiratory infection, favored by tropism, encourages SARS‐CoV‐2 self‐assembly at scale and is a fundamental driver of global spread. In contrast, lower respiratory tract infection does not favor SARS‐CoV‐2 self‐assembly at scale but critically drives COVID‐19 pathophysiology. It is this pathophysiology that is thoroughly explored by Lumbers et al.^5^ in their sequenced review.

COVID‐19 has reminded us that coronaviruses are a particularly interesting example of increased virulence associated with the emergence of novel viruses, because of the very essential role that its receptor for cell entry plays in controlling inflammation and the immune response. SARS, SARS‐Cov‐2, and HLN63 are three coronaviruses that use the angiotensin‐converting enzyme receptor type II (ACE2) to enter cells. All three viruses cause morbidity and mortality via an overwhelming immune response, mediated at least in part by angiotensin II. SARS‐Cov‐2 has the highest affinity for the ACE2 receptor and HLN63 the lowest. All three coronaviruses enter the body via the nasopharynx or gastrointestinal tract because these parts of the body have a high density of ACE2 receptors. Nasopharyngeal colonization and secretions can lead to introduction of the virus into the lungs and the risk of severe respiratory infection due to the destruction of respiratory ACE2 receptors on binding SARS‐CoV‐2.

In fact, as outlined by Lumbers et al. in this Themed Review^5^ it is critical in understanding the pathogenesis of COVID‐19 to be aware of the destruction of the ACE2 receptor induced by SARS‐CoV‐ 2 binding. ACE2 is a component of the renin–angiotensin system (RAS) which is classically viewed as a circulating endocrine system which through the actions of its major peptide, angiotensin II (Ang II) controls blood pressure and fluid and electrolyte homeostasis. Drugs that limit the actions of Ang II either by limiting its production or interfering with its interaction with the AT1 receptor have therefore been a mainstay in the treatment of hypertension and cardiac fibrosis. What is less widely appreciated is the role of local RAS in various organs and tissues, including fat tissue and vital organs.^6^ However, the value of drugs that block the RAS in preventing organ damage is most evident in the heart and kidneys. The term “tissue RAS” may, however, limit our thinking. Aldosterone and its resultant endothelial damage, vasoconstriction and fluid retention is also implicated in fibrotic disease from COVID‐19 infection.

It also appears that ***tissue*** angiotensin peptides and anti‐aldosterone agents would seem to be a key focus of interest in discussing repurposing of drugs to treat inflammation induced by SARS‐CoV‐2 infection^5^ rather than considering the RAS in its classical *circulating mode*. From a therapeutics perspective and as Lumbers et al. detail, there are three pathways via which angiotensin peptides can be prevented from acting on the AT1R—block their formation, block binding to the AT1R, or enhance Ang II metabolism by those proteases that do not lead to the production of Ang II mimicking peptides (Ang III and Ang IV). Angiotensin‐converting enzyme inhibitors (ACEi) block the formation of Ang II from Ang I. Angiotensin receptor blocking (ARB) drugs block the actions of Ang II via its AT1R.

Additionally, the enzyme ACE 2, a homologue of ACE, has two critical actions in blocking Ang II which are also potential key targets for therapeutics development. First, it catabolizes Ang II to Ang (1‐7) and in doing so, it produces Ang (‐1‐7) which has opposing effects on tissues, cells, biochemical pathways etc, to those produced by Ang II. Like ACE, ACE2 is widely distributed throughout almost every organ of the body and along the lining of blood vessels. Furthermore, ACE2 is a highly conserved protein having appeared early in evolution as it is found in chordates >100 million years ago. All examples of human viruses with broad host ranges use highly conserved cell receptors (i.e., with more than 90% amino acid sequence homology).

There are thus major consequences of destruction of ACE2 by SARS‐CoV‐2 including enhanced activity of tissue angiotensins and extremely widespread involvement of tissues and organs, and a multiplicity of effects causing inflammation that can be modified by the severity of infection, immune regulation/dysregulation. Lumbers et al. also elegantly show how SARS‐CoV‐2 by interacting with its receptor ACE2, turns the host response to infection into a dysregulated uncontrolled inflammatory response. It seems clear, from a drug development pathway, that blockade of the actions of Ang II must be seriously considered to provide a therapeutic pathway for patient treatment in COVID‐19.

In retrospect, it is clear that infection with SARS‐CoV‐2 (COVID‐19) swept the world with amazing rapidity in contrast to the related SARS and MERS. Its major threat was to overwhelm universal health care systems particularly because of the number of older and comorbid patients affected, although it does not spare the young or healthy from developing severe forms of the disease requiring prolonged intensive care. Regardless of whether infection is asymptomatic or not, SARS‐CoV‐2 causes long‐term sequelae (including so‐called “long COVID”) which is starting to appear to impose considerable health and economic burden for future generations.^7^

Therefore, in this Journal, Jarrott et al.^8^ has teased apart “Long COVID,” both to develop a hypothesis for understanding the biological basis of the differential host response, and to guide clinical trials of pharmacological treatment strategies. It is noted that a significant proportion of people experience this “Long COVID”—a variety of troubling symptoms that appear to be associated with persistent low‐level cytokine concentrations and low‐grade general inflammation. Among other therapeutics that can affects these pathways, drugs that activate the intracellular transcription factor nuclear factor erythroid‐derived 2‐like 2 (Nrf2) may increase the expression of enzymes to synthesize the intracellular antioxidant glutathione that will quench the free radicals that cause oxidative stress. Interestingly, Jarrott suggests that the hormone melatonin, already a therapeutic in clinical use, has been identified as an activator of Nrf2 and it is discussed as a potential pharmacological treatment option, to be studied in clinical trials.^8^

This Themed Review thus aims to fill the gap of understanding the therapeutics targets for drug development in COVID‐19 by taking a detailed overview of the evolutionary behavior of the virus, the viral and human environment, the immune response of mammals (and more specifically humans), to coronaviruses. It teases out the simple yet complex interweaving of physiology and immunology together with the basic laws of thermodynamics which drive viral entry. Only with this knowledge can pharmacologists develop reasonable therapeutic approaches to study in more depth, obtain administrative data on current use and in some cases move to a clinical trial. Although this series stops short of providing a complete suite of therapies for COVID‐19 treatment, knowledge of the basic science and related systems enables pharmacologists to move forward. It enables a rational approach to the consideration of therapeutics—the individualized drug, the dose, the timing, and the combination in the treatment of this and future pandemics.

Readers of this Themed Review will see that the movement of SARS‐CoV‐2 within humans and across the human populations displays the key characteristics of a complex system that underpins COVID‐19 disease. That is, it is a powerful motive for reflecting on potential for therapeutic development, as timing and dose is key. Furthermore, this complex system is powerfully described by the thermodynamic considerations underpinning the physiological and pharmacological properties of SARS‐CoV‐2, from infection of a single cell to its global spread in this pandemic. We must accept that the virus is the metaphorical center of our biological solar system and humans merely an opportunity for it to replicate at scale and to mutate. Underlying this is the opportunity to find and exploit the weaknesses of the virus, as well as halt the inflammatory process, driven through Ang II becoming a vasoconstrictive endothelialitis. This knowledge will enable a scientific basis by which to choose the best drugs for clinical trialing and in the appropriate dosages and timing.

Collectively the three reviews provide a time‐based portrayal of COVID‐19 disease from its first entry to the human to its damaging deregulation of the inflammatory response to the devasting prolonged effects with “Long COVID.” Based on a systems approach made possible by a pharmacological approach, the reviews integrate the important underpinning descriptive sciences, described for COVID‐19 disease. In this way, a dynamic view of the viral infection, passage, and pathophysiology is obtained. Importantly these reviews addressed the issue of repurposed therapeutics in the context of the pathogenic responses to COVID‐19 disease as previously highlighted.^1^ This is made possible by the unique features of human physiology, pathology, and pharmacology; the disciplines that permit an integrated systems analysis approach to this disease. It will be important for the future to project system analysis with these disciplines to the forefront of responses to further unforeseen subsequent pandemics.

## DISCLOSURE

The authors declare no conflict of interest.

## AUTHORS’ CONTRIBUTIONS

Both Professor Jennifer Martin and E/Professor Richard Head contributed equally to this Editorial and the guidance of the manuscripts in this Themed Issue.

## Data Availability

There are no data to share in this Editorial and the authors would be delighted to engage with readers in conversation with the topics raised in this Themed review.
